# Ethnic Enclaves, Economic and Political Threat: An Investigation With the European Social Survey

**DOI:** 10.3389/fsoc.2021.660378

**Published:** 2021-07-06

**Authors:** Neli Demireva, Wouter Zwysen

**Affiliations:** ^1^ Department of Sociology, University of Essex, Colchester, United Kingdom; ^2^ European Trade Union Institute, Brussels, Belgium

**Keywords:** ethnic enclave, local area, economic threat, political threat, majority, migrants, minorities

## Abstract

This article examines the labor market outcomes and political preferences of majority, minority, or migrant individuals who report that they live in an ethnic enclave—a neighborhood with few majority residents. Politicians often proclaim that ethnic enclaves are problematic, but there is little rigorous examination of these claims. The ethnic composition of a local residential area can affect its inhabitants negatively by increasing conflict and competition (real or perceived) between groups. Majority members may feel their economic and political power questioned and think that the resources to which they are entitled have been usurped by newcomers. Migrants and minorities can be negatively impacted by isolation from the mainstream society, and their integration attempts can be hindered in ethnically concentrated local areas. Using data from the 2002 and 2014 waves of the European Social Survey, enriched with contextual data, we examine the impact of ethnic enclaves accounting for selection and compositional differences. We do not find evidence that minority concentrated areas impact negatively upon the economic outcomes of majority members, not even of those in precarious positions. We do however find that residence in enclaves is associated with greater propensity to vote for the far right and dissatisfaction with democracy for the majority group. Furthermore, there is an economic enclave penalty associated with the labor market insertion of migrants and the job quality of the second generation, and ethnic enclaves also increase the dissatisfaction with democracy among the second generation. We discuss our findings in light of the threat and contact literature.

## Introduction

Increased migration to and within Europe has made ethnic diversity in local areas an everyday experience for majority populations across Europe (Iceland, 2014; Laméris et al., 2018). While residential segregation is not as high as in the United States, migrants and their children often live concentrated in more deprived areas than the majority (Semyonov et al., 2012). Such spatial local concentration of migrants and minorities is often referred to as an ethnic enclave (Portes and Zhou, 1993; Waldinger, 1994) and is interpreted by politicians as problematic, hindering integration for minorities and increasing their sense of distance from the mainstream (Cameron, 2011).

While important, little is known empirically about the impact of residential isolation from the mainstream society. In this article, we use cross-nationally comparable European data to study whether there is an association between the perceived ethnic composition in the local residential area and two sets of outcomes: on one hand, labor market outcomes, and on the other hand, political behavior. In this way, we capture the distinction between threat to labor market position (heightened competition should result in observed poor labor market outcomes) and threat to political power (which should result in greater support of far-right parties which programs frequently stoke such fears, or higher salience of political nationalism and dissatisfaction with democracy).

We contribute to the literature first by comparing how ethnic enclaves affect majority members as well as migrants and minorities and by providing valuable insights into the conflict and contact mechanisms at work. Second, we make use of propensity score matching methods to account directly for selection into local areas as selection on observables can be a major part of the story. Third, we account for the influence of important mediating factors such as the regional employment rate, the friendship contacts of our respondents, and neighborhood social disorganization captured by fear of crime. This study relies on a measure of the perceived ethnic composition of the local area. One limitation of our data is the possible variability in subjective perceptions of the area’s residential composition. Nevertheless, previous research has demonstrated that perceptions matter—it is the perceived rather than the actual size of the minority population that is more likely to increase anti-minority sentiment (Semyonov et al., 2012). We elaborate further on the limitations of this work in the Discussion section.

## The Effect of Ethnic Composition of Local Area

### Selection Into Ethnic Enclaves

Migrants and minorities may concentrate in less-desirable areas as they are cheaper, through a higher reliance on social housing (Semyonov and Glikman, 2009), or can be pushed there by housing discrimination (Boeri et al., 2015). Migrants and minorities may also seek out ethnic enclaves as they can offer shelter from discrimination and access to ethnic goods and positive social and cultural connections (Portes and Zhou, 1993; Zhou, 1994; Zhou, 2005; Bécares et al., 2009). These areas, however, are often more deprived and can provide fewer good job prospects (Feng et al., 2015). It is therefore important to account for selection into ethnic enclaves when estimating their impact (Edin et al., 2003; Damm, 2009; Andersson et al., 2013; Boeri et al., 2015).

### The Threat to Economic or Political Power and the Ethnic Enclave

The ethnic composition of a local residential area can affect its inhabitants negatively by increasing conflict and competition (real or perceived) between groups (Blalock, 1967; Glikman and Semyonov, 2012). Majority members can feel their economic and political power questioned and think that the resources to which they are entitled have been usurped by newcomers (Vervoort, 2012). In this study, we focus on both dimensions and examine the impact of the ethnic enclave on labor market outcomes (economic position) and vote for far-right parties, feeling close to the far right and dissatisfaction with democracies (which demonstrates the salience of political nationalism observed when political power is threatened).

There is inconclusive evidence of whether there is labor market competition between migrants and majority members. Some studies find a somewhat negative effect of increasing migration on earnings and employment of the majority (Dustmann et al., 2013; Bratsberg et al., 2014; Brücker et al., 2014); while others find positive or no effects (Bellini et al., 2013; Akay et al., 2014; Dustmann and Frattini, 2014; Aleksynska and Tritah, 2015; Ehrlich and Kim, 2015; Peri et al., 2015; Mayda et al., 2018). While competition is mainly measured at the regional level, there is some evidence at the local level (primarily based on United Kingdom data) for increased competition between migrants and majority members, with migrants affecting majority members’ employment probability negatively (Dustmann et al., 2017). Boeri et al. (2015) show a negative effect of the extra local area competition on migrants in Italy. Zwysen and Demireva (2020) show that the presence of migrants does not really lead to worsening of the job quality of majority members, but there are adverse effects for migrants and some minority groups.

Is there an association between residing in ethnic enclaves and political behavior? The research on this topic is also somewhat contradictory. Pardos-Pardo et al. (2014) found evidence that concerns over immigration strengthen the identification of majority members with the center-right party that owns this immigration issue. Halla et al. (2017), using Austrian data, reported a strong association between increasing immigrant influx in a community and the voting share for the Freedom Party of Austria. The greater presence of migrants becomes associated with the economic concerns of the majority for their labor market position on one hand and with fear for access to amenities on the other. Most recently, concerns have shifted to refugees (Dinas et al., 2019; Dustmann et al., 2019). Unfortunately, our data do not allow us to identify who majority members perceive as an outgrouper. Moreover, despite the economic benefits of migration, majority members may see either their economic position threatened by mass migration (Becker and Fetzer, 2016) or their political power challenged and seriously eroded (Tabellini, 2020). Thus, there are several indications that the ethnic enclave can bring about discontent on the part of majority members. First of all, according to the threat framework, the ethnic enclave will increase the proximity between different ethnic groups, while at the same time majority members constitute a minority in this setting (Kawalerowicz, 2021). Second, enclaves are also characterized by higher levels of structural disadvantage and economic insecurity, and deprivation may act to strengthen identification with populist projects (Margalit, 2019) and opposition to outgroupers (Hjerm, 2009; Finseraas and Kotsadam, 2017). Left-behind voters can become disillusioned with the democratic process and react in relation to broader grievances that are associated with their changed economic position over time rather than with the presence of migrants or minority members in their local area (Hjerm, 2009; Matti and Zhou, 2017; Haaland and Roth, 2020). Whereas this study examines a variety of mediating factors and looks at the outcomes and preferences of majority individuals in precarious economic positions, our analysis does not allow us to examine changes to local areas over time either in terms of deprivation or minority composition and identify respondents who can be considered “left behind.” Finally, studies have shown that settled migrants and minorities can also have strong reservations against migrants and may act to preserve their established positions (Just and Anderson, 2015).

Research Expectation 1: Salience of Economic Threat. The residence in the ethnic enclave can bring worse labor market outcomes for majority members (if there is competition in the local labor market). Greater non-majority presence in the local area may also align with worsening of the labor market position of first- and second-generation individuals as the pool of workers substitutable with one another directly increases.

Research Expectation 2: Salience of the Threat to Political Power. The residence in the ethnic enclave may be associated with greater propensity to vote for the far right for majority members (if there is conflict). Similar outcomes can be witnessed among the second generation, whose position can also be threatened by the growing presence of migrants. As migrants frequently do not possess political power, the threat to them should be weaker.

Conflict and threat can be important mediators of the relationship between the enclave and the outcomes in our study. We use several measures of conflict to capture this mechanism. Fear of crime (a subjective measure of conflict) can be strong in areas which majority members perceive as dominated by outgroupers (individuals belonging to an ethnic group other than their own) as shown by studies both in Europe and the United States (Semyonov et al., 2012; Laméris et al., 2018). Crime levels are usually lower in areas with greater presence of migrants (Han and Piquero, 2021), but we also control for whether the respondent reports being a victim of crime (an objective measure of conflict). Finally, the economic deprivation of the region and the respondent’s personal precarious economic position have been linked to anti-minority sentiments and the ability to cope with adversity (Haaland and Roth, 2020; Kawalerowicz, 2021). Using these measures, we formulate the following research expectation.

Research Expectation 3: *Conflict Mechanism*: If the ethnic enclave is characterized by higher levels of conflict and economic hardship, a negative impact of the local area will be observed that is not due to its ethnic homogeneity but to concentrated disadvantage and conflict. We investigate the role of several mediating factors often considered as part of the threat framework.

Furthermore, individuals who are in a weaker or precarious economic position may be particularly susceptible to narratives of opposition between majorities and migrants and minorities (Haaland and Roth, 2020) which will express itself in identification with far-right projects and dissatisfaction with democracy. We investigate whether the threat to political power is mediated by the economic position of the individual—whether they are employed or not, or by the quality of their jobs.

### The Contact Mechanism

Enclaves may also affect the economic and political power of different groups by shaping the ties respondents form, particularly of migrants and minorities who would be more likely to form contacts with other minorities but have fewer opportunities to engage in bridging ties with the majority, especially if they reside in an enclave (Vervoort et al., 2012; Danzer and Yaman, 2013). Areas in which encounters with the majority are few may lead to a further rejection of outgroupers on both sides (Vervoort et al., 2012; Skvoretz, 2013). Tie formation strongly affects the labor market outcomes of migrants and second-generation individuals as has been shown by previous research. Bridging ties foster further sociocultural integration of migrants by improving language skills and can be particularly important to second-generation minority members, as bridging may affect the sharing of information and the presence of useful social networks (Chiswick, 2009; Semyonov and Glikman, 2009; Lancee and Hartung, 2012; Vervoort, 2012; Danzer and Yaman, 2013). Lack of bridging ties in the second generation is seen as particularly problematic by policymakers (Cameron, 2011) and may signal the existence of long-term inclusion issues (Koopmans, 2010).

Research Expectation 4: Importance of Ties for Labor Market Outcomes. We do not expect bridging contacts to impact the labor market position of majority members, but the previous literature suggests that they are crucial for the successful economic insertion of migrants and the second generation. Lack of such bridging ties may be responsible for the observed economic enclave penalty among migrants and the second generation.

Research Expectation 5: Importance of Ties for Political Outcomes. Bridging ties to outgroupers may act to reduce feelings of social distance on the part of majority members and can be positively associated with satisfaction with democracy and negatively associated with voting for the far right. Bonding among migrants and minorities may represent a reaction to defend ethnic boundaries, especially under conditions of discrimination. We should see then that migrant and minority bonding ties are associated with reduced likelihood to vote for far-right projects which usually position themselves in conflict with these groups.

There can also be important differences between migrants and the second generation. Established groups born in the immigrant society may feel that their interests align closely with those of majority members and may also fear exposure to competition with migrants (Martin, 2015). Migrants may also benefit more from possible support within the ethnic enclave, while this is less the case for the second generation. Our study is well-placed to explore these differences.

## Data and Empirical Strategy

### Data and Main Concepts

#### Outcomes

We study three main labor market outcomes to capture threat to economic power: activity, employment, and job quality measured as occupational status (Ganzeboom et al., 1992). We use three main variables to measure threat to political power: first, whether someone voted for a right-wing nationalist party in the last national elections; second, whether someone feels closest to a right-wing party. We follow the work by Eger and Valdez (2015) in classifying parties.^1^ As a third measure, we include an 11-point scale of satisfaction with democracy in the country of survey, ranging from extremely dissatisfied to extremely satisfied. We consider these outcomes for first-generation migrants, second-generation individuals, and majority members separately.^2^ We use the European Social Survey (ESS), a cross-national representative dataset which included modules on immigration in 2002^3^ and 2014^4^ and include the EU-15 member states as well as Norway.^5^ Migrants are defined as respondents born in another country and with at least one parent born abroad; the second-generation individuals are defined as those born in the country of residence with at least one parent born abroad; and majority members are themselves born in the country as well as their parents. We exclude majority members who self-report as an ethnic minority and restrict the sample to those aged 16–60 years who are not in education or retired. After listwise deletion of missing cases, the sample consists of 28,508 respondents, of whom 2,421 are first-generation migrants and 1,842 are second generation.

#### Defining the Ethnic Enclave

In 2002 and 2014, respondents were asked how they would describe the local area where they currently live^6^ in terms of the presence of members of a minority race or ethnic group with the following answer categories: almost none, some, or many. We dichotomize this variable to distinguish respondents living in an ethnic enclave—an area with high concentration of migrant and minorities from those with some or almost none. Around 10% of the majority, 25% of migrants, and 19% of the second-generation individuals live in these ethnic enclaves.

Using self-reported presence of minorities brings the risk that the perception of minority presence differs across individuals, and the same area may be classified differently by different people. Problematically, minority presence may be perceived to be higher by those for whom it is seen as an issue. While we would ideally use the share of migrants or minorities in the local area, no cross-nationally comparative European data exist at the local level. Descriptively, we can compare the regional reported average of living in an ethnic enclave to the actual share of first- and second-generation individuals in a region (NUTS-1 and NUTS-2: much higher than the perceived local area), obtained from the EU Labour Force Survey (LFS) ad hoc modules of 2008 and 2014, as shown in Supplementary Figure A1 in the supplementary material. This does indeed show that the probability of a respondent living in an ethnic enclave is higher in regions with objectively more migrants and second-generation individuals (*r* = 0.56).

Variants of this measure were used to study the relation between residential concentration of minorities and contact, migrant attitudes, and feelings of safety (Semyonov and Glikman, 2009; Semyonov et al., 2012). It is clear that while there is subjectivity in this measure, it is particularly important to consider subjective perceptions when estimating the impact of residing in such local areas; and it is also clear that there is a link to the objective reality.

Descriptive statistics of all variables can be found in Supplementary Table A1 in the supplementary material.

### Methodological Strategy

#### Main Effect

We cannot randomly assign individuals to live in an ethnic enclave. Instead, we can study the difference in outcomes that are observed for an individual living in such an enclave and an individual who is not but is otherwise very similar by matching them on a range of covariates (that are likely to have driven residential selection in the first place).^7^ First, we estimate the effect (Δ) of living in an ethnic enclave on economic position or threat to political power after accounting for selection into these areas—this estimate is hereafter designed the “treatment.” The effect is the difference between the outcome y when living in an ethnic enclave T (y_1_) and when not living in the enclave (y_0_) as shown in Eq. 2. As both outcomes cannot be observed simultaneously, the potential outcomes are estimated using propensity score matching (Cameron and Trivedi, 2005). We estimate the effect of living in the enclave separately for majority members, migrants, and minorities.

$$△=\left({\text{y}}_{1}\text{ | T}=1\right)-\left({\text{y}}_{\text{0}}\text{ | T}=\text{0}\right)$$ (1)

p(x) = Pr(T=1 | X=x) (2)

The propensity score p(x) indicates the probability of living in an ethnic enclave conditional on the observed characteristics X, as shown in Eq. 2 (Caliendo and Kopeinig, 2008). In order to compare like with like, we include year of survey, country of residence, and dummies for living in a big city; the outskirts or suburbs of a big city, a town, or small city, or in a more rural environment; and sociodemographic characteristics that can affect labor market outcomes and selection into localities (highest qualification, gender, age, marital and family situation, an indicator of having poor health, the highest qualification obtained by a parent, occupational class of the highest-status parent when the respondent was aged 14 years, and a dummy indicating whether the respondent lived with both parents at age 14 years). For migrants and the second generation, we also include a dummy indicating whether one of their parents is born in the host country. For migrants, the years of residence are also included.^8^

Several matching algorithms were tested (not shown here), and the best balance overall as well as an acceptable match for all instances was obtained by matching on five nearest neighbors with replacement.

#### Mechanisms (the Effect of Mediators)

In a second step, we include proxies for conflict and contact to the variables on which individuals are matched. This means that we are comparing people who live in an ethnic enclave with their counterparts who do not, but who have similar levels of local conflict, or of contact with majority and minority members. The extent to which this changes the estimated treatment effect can indicate whether these mechanisms play a role in the observed differences.

*Conflict* is measured through factors that can indicate strife and competition. First, we include the employment rate at the regional level^9^ estimated through the EU Labour Force Survey (LFS) in 2002 and 2014 to account for resources in the local labor market, a commonly adopted measure in economic studies (Manacorda et al., 2012), and overall deprivation. The region is much larger than the locality, but it does provide some indication of differences in resources and opportunities across the sample. Second, we include a dummy variable indicating that the respondent was a victim of burglary or assault in the last 5 years—crime being an oft-cited indicator of conflict in the literature, and third is a dummy indicating whether respondents feel unsafe walking alone at night in their local area.

*Contact* is approximated by including a dummy variable indicating whether respondents speak the host country language as their main language to proxy sociocultural integration and by a question measuring close friendships with migrants/minorities.^10^ The question on language varies very little among the majority, but this mechanism would not be expected to affect them.

Furthermore, in order to study whether the association between living in an ethnic enclave and political outcomes is due to economic uncertainty and deprivation, we test whether matching workers on an indicator of their economic outcomes—being active, being employed in low-status jobs (lowest 25% of occupational status), being employed in middle-status jobs (middle 50% of occupational status), or being employed in high-status jobs (highest 25% of occupational status)—produces an effect on the relationship.

#### Sensitivity Tests

We test the robustness of our findings, including their sensitivity to unobserved confounders. While the propensity score matching accounts for selection on observed characteristics, there may be selection on unobserved characteristics such as motivation or preferences which can affect both the probability of living in an ethnic enclave and labor market outcomes, and thereby bias the estimated effect. We test the robustness of these results to three simulated unobserved binary confounders (Rosenbaum, 2005; Nannicini, 2007), mimicking the relations of three strong confounders: having tertiary qualifications, feeling unsafe when walking in the local area at night, and a self-reported measure on whether the household struggles financially. Our overall findings are supported; more results can be found Supplementary Tables A4–A6 in Supplementary Appendix S1 in the supplemental material.

We further estimate the difference between living in an ethnic enclave and living outside of an ethnic enclave when using different specifications of the matching process.

## Results: Threat to Economic Position and Political Power

### Descriptive Results

Table 1 shows the differences between individuals residing in the ethnic enclave and those outside it. Notably, across the three ethnic categorizations we consider majorities, migrants, and the second generation, those who are living in the enclave are doing worse in terms of their labor market outcome, while being more likely to vote for the far right, identify with the far right, and be satisfied with democracy. Majority and minority members are very similar in terms of level of activity and employment, and minority members have better status. Migrants have worse employment outcomes, than majority or second-generation members while they are much less likely to vote for the far right and have more satisfaction with democracy. There is clear evidence of bonding in the ethnic enclave for both the first generation and for minority individuals and reduced likelihood to adopt the language of the country of origin as the main language spoken at home.

**TABLE 1:** Average labor market and political outcomes for those living in ethnic enclave and those who do not.

	Majority		Migrants		Second generation	
	Not in enclave	Ethnic enclave	Not in enclave	Ethnic enclave	Not in enclave	Ethnic enclave
Active	0.90 (0.30)	0.88 (0.32)	0.88 (0.32)	0.83 (0.38)	0.90 (0.30)	0.90 (0.30)
Employed	0.92 (0.27)	0.88 (0.33)	0.89 (0.32)	0.8 (0.40)	0.92 (0.28)	0.82 (0.39)
Occupational status	46.82 (18.88)	46.16 (19.11)	43 (20.61)	40.74 (20.05)	48.47 (18.8)	43.67 (19.16)
Vote far right	0.06 (0.24)	0.09 (0.29)	0.01 (0.11)	0.03 (0.16)	0.05 (0.23)	0.05 (0.23)
Feel close to far right	0.06 (0.23)	0.08 (0.27)	0.04 (0.2)	0.04 (0.2)	0.07 (0.25)	0.09 (0.28)
Satisfied with democracy	5.61 (2.33)	5.01 (2.56)	6.36 (2.36)	6.1 (2.48)	5.64 (2.39)	5.07 (2.56)
Feel unsafe when walking in local area after dark	0.14 (0.35)	0.33 (0.47)	0.15 (0.36)	0.31 (0.46)	0.17 (0.37)	0.34 (0.47)
Victim of burglary/assault last 5 years	0.22 (0.42)	0.3 (0.46)	0.22 (0.42)	0.24 (0.43)	0.24 (0.43)	0.33 (0.47)
Employment rate in regional area (centered)	0.08 (5.17)	−1.12 (5.85)	0.48 (4.65)	−0.82 (5)	0.85 (3.83)	0.05 (4.51)
Several immigrant/minority friends	0.12 (0.33)	0.21 (0.4)	0.48 (0.5)	0.58 (0.49)	0.25 (0.44)	0.47 (0.5)
A few immigrant/minority friends	0.39 (0.49)	0.41 (0.49)	0.35 (0.48)	0.31 (0.46)	0.44 (0.5)	0.33 (0.47)
No immigrant/minority friends	0.49 (0.5)	0.38 (0.49)	0.17 (0.37)	0.11 (0.32)	0.31 (0.46)	0.2 (0.4)
Speak country language at home	0.98 (0.15)	0.97 (0.16)	0.57 (0.49)	0.45 (0.5)	0.94 (0.23)	0.88 (0.32)

Note: The table shows the average and standard deviation of labor market outcomes and political outcomes, by origin and whether they live in an ethnic enclave.

### Economic Enclave Penalty

#### Mean and Matched Difference: Threat to Economic Position

Table 2 compares the average difference in activity, employment, and occupational status between those living in an ethnic enclave and their counterparts (mean difference), with the treatment estimate after matching (matched difference). Residents of ethnic enclaves are on average less likely to be active, 5–10 percentage points less likely to be employed, and migrants and especially second-generation individuals living in enclaves also work on low-quality jobs on average than those living in mixed or majority-dominated areas.

**TABLE 2:** Difference in labor market activity, employment status, and occupational status of inhabitants of ethnic enclaves and those in majority areas.


		Majority	Migrants	Second generation
Active (%)	Mean difference	−1.55** (0.65)	−4.88*** (1.59)	−0.59 (1.77)
	Matched difference	−0.22 (0.72)	−3.46** (1.75)	−2.32 (1.65)
	N treated | control	2,441 | 21,721	596 | 1807	345 | 1486
Employed (%)	Mean difference	−4.55*** (0.62)	−8.927 (1.74)	−9.98*** (1.9)
	Matched difference	−1.68** (0.8)	−6.69*** (2.12)	−.58 (2.86)
	N treated | control	2,152 | 19,487	496 | 1592	310 | 1344
Status	Mean difference	−0.68 (0.46)	−2.36** (1.17)	−5.07*** (1.31)
	Matched difference	−0.63 (0.44)	−0.84 (1.09)	−2.56** (1.24)
	N treated | control	1864 | 17,692	393 | 1392	250 | 1218

*: *p* < 0.1; **: *p* < 0.05; ***: *p* < 0.01; The mean difference is estimated through an independent samples *t*-test (two-tailed), and the matched difference shows the estimate after propensity score matching with five nearest neighbors, taking individual sociodemographic factors into account.

Selection clearly matters and is important to take into account, as the inhabitants of ethnic enclaves are generally lower educated, female, less healthy, young, and of lower parental social class. Living in an ethnic enclave is also much more likely for residents of big cities than in small towns or rural areas. We find that matching on the propensity score resulted in good balance of covariates between the enclave residents and non-enclave residents.^11^ Supplementary Table A2 in the supplementary material shows the selection equation, and Supplementary Table A3 shows the averages of the covariates before and after matching.

**FIGURE 1:**
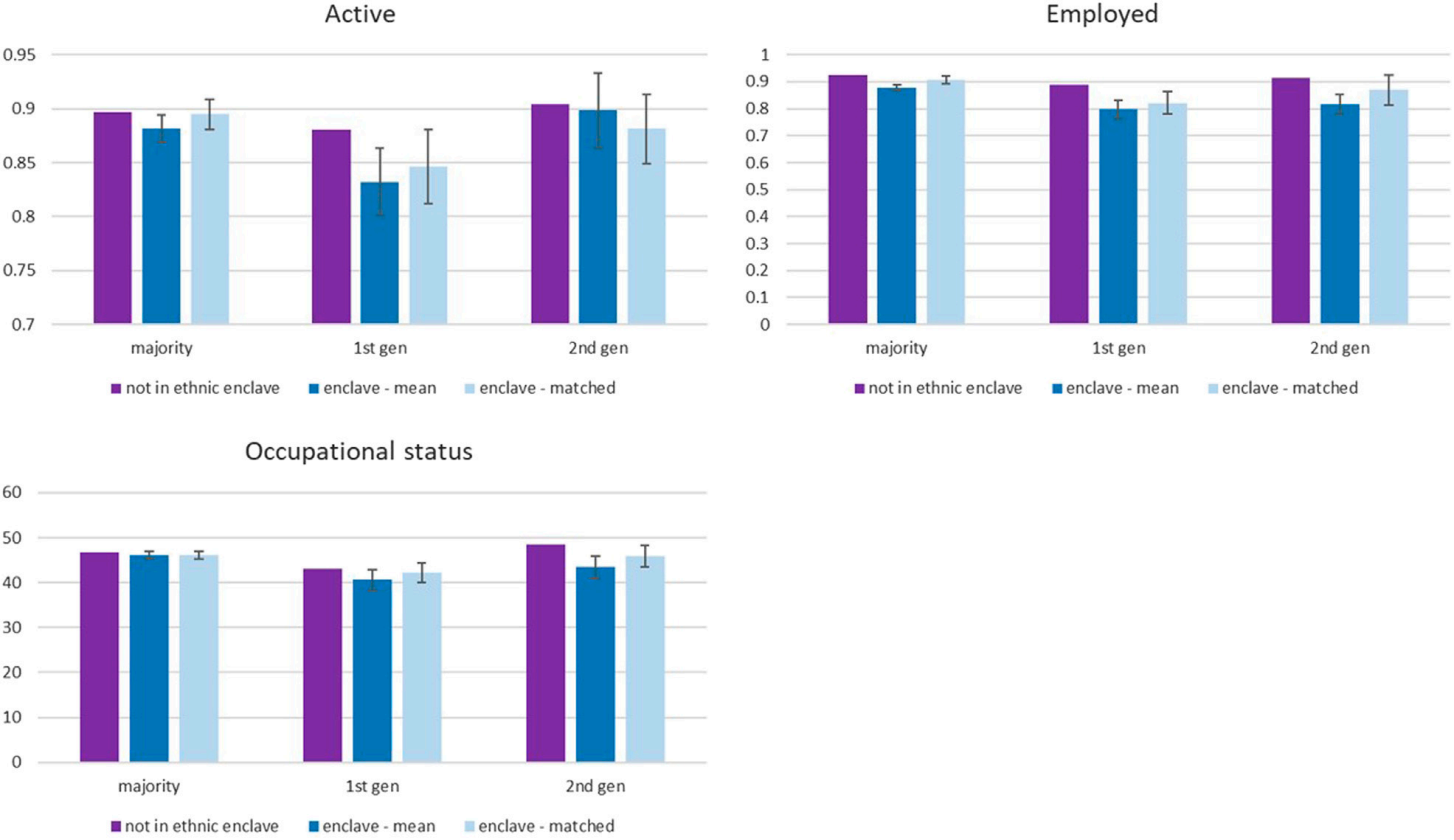
Labor market status for inhabitants of an ethnic enclave and those living elsewhere—by migrant status. The mean difference is estimated through an independent samples *t*-test (two-tailed), and the matched difference shows the estimate after propensity score matching with five nearest neighbors, taking individual sociodemographic factors into account. Outcome variables activity, employment, and occupational status are examined and the difference between enclave and non-enclave residents reported.

After accounting for selection on observables between respondents, the estimated differences between residents and nonresidents of enclaves diminish (see Table 2). Particularly, for majority members, the negative association of living in an ethnic enclave with one’s economic position is to a large extent driven by a range of other factors such as individual sociodemographics, but we still observe slightly poorer employment prospects for majority members. In contrast, Figure 1 shows that substantial negative effects remain for migrants after the matching: Migrants residing in ethnic enclaves are 3.5 percentage points less likely to be active and almost 7 percentage points less likely to be employed, while second-generation individuals work on lower quality jobs (occupational status reduces by 2.5 points, which is a 5% reduction in status relative to the average occupational status [48.5] of second-generation individuals living outside of ethnic enclaves).

Living in an ethnic enclave does not seem to threaten the economic power of majority members very strongly, while our results align with interpretations of competition within the migrant pool for jobs and in terms of job quality for the second generation.

#### Mediation: Conflict and Contact in Relation to Economic Position

In this section, we test our conflict and contact hypotheses. Table 2 shows how the difference between those living in an ethnic enclave and their counterparts who do not changes when we also compare them to people experiencing a similar level of conflict or contact.^12^

The first column of Table 3 shows that majority members who are living in an ethnic enclave are 1.7 percentage points less likely to be employed than non-enclave majority members. This difference is not affected much when accounting for conflict or contact with minorities. This result is not driven by poorer regional employment prospects or feelings of threat. There is no impact on activity or occupational status.

**TABLE 3:** Conflict and contact mediating the relationship between ethnic enclaves and labor market outcomes.

		Majority	First generation	Second generation
Active (%)	Effect of ethnic enclave	−0.22 (0.72)	−3.46** (1.75)	−2.32 (1.65)
	When accounting for conflict	−0.05 (0.74)	−3.46* (1.9)	−3.25* (1.67)
	When accounting for contact	−0.45 (0.73)	−2.62 (2.01)	−2.03 (1.72)
Employed (%)	Effect of ethnic enclave	−1.68** (0.8)	−6.69*** (2.12)	−4.58 (2.86)
	When accounting for conflict	−1.82** (0.83)	−6.13*** (2.19)	−3.03 (2.77)
	When accounting for contact	−1.86** (0.81)	−5.08** (2.16)	−6.39** (2.64)
Status	Effect of ethnic enclave	−0.63 (0.44)	−0.84 (1.09)	−2.56** (1.24)
	When accounting for conflict	−0.89** (0.45)	0.24 (1.1)	−3.53*** (1.27)
	When accounting for contact	−0.68 (0.44)	−0.01 (1.03)	−3.19** (1.39)

Note: This shows the estimated difference between those living in an ethnic enclave and their counterparts who live in a more homogeneously majority area. People are matched on country and sociodemographic background. To account for conflict, we include feelings of unsafety, being a victim of crime, and regional unemployment rate. To account for contact, we include friendshipties with minority members and language skills.*: *p* < 0.1, **: *p* < 0.05, ***: *p* < 0.01.

Migrants who live in an ethnic enclave are less likely to be active (3.5 percentage points) and less likely to be employed than their counterparts who live in an area with more majority members. These differences seem partly driven by migrants having fewer contacts with the majority—meaning more minority friends and speaking the language less well—than their counterparts who live elsewhere. When accounting for contact, the difference in activity is reduced by 1 percentage point and no longer statistically significant, while the employment gap is reduced by 1.5 percentage point. The employment gap is also driven by conflict, as it was for the majority.

Finally, for the second generation, we see that contact does not explain their worse quality jobs and lower employment probability. Accounting for conflict in the area does seem to account for around 30% of the gap in occupational status.

There have been concerns that in line with classic conflict literature (Bobo, 1988), conflict may operate asymmetrically. Majority members who have more to lose in terms of their political and economic power should be more susceptible to conflict, particularly to concerns about crime. Yet, we find an economic enclave penalty however not for majority members but for migrants and minority members after taking into account mediators such as conflict and contact. Our results suggest that living in an ethnic enclave is particularly bad for migrants’ employment and for the job quality prospects of the second generation, and this can be exacerbated through isolation from the mainstream due to the formation of primarily bonding ties. We next turn to threat to political power that the enclave can posit.

### Political Enclave Penalty

#### Mean and Matched Differences: Threat to Political Power

**FIGURE 2:**
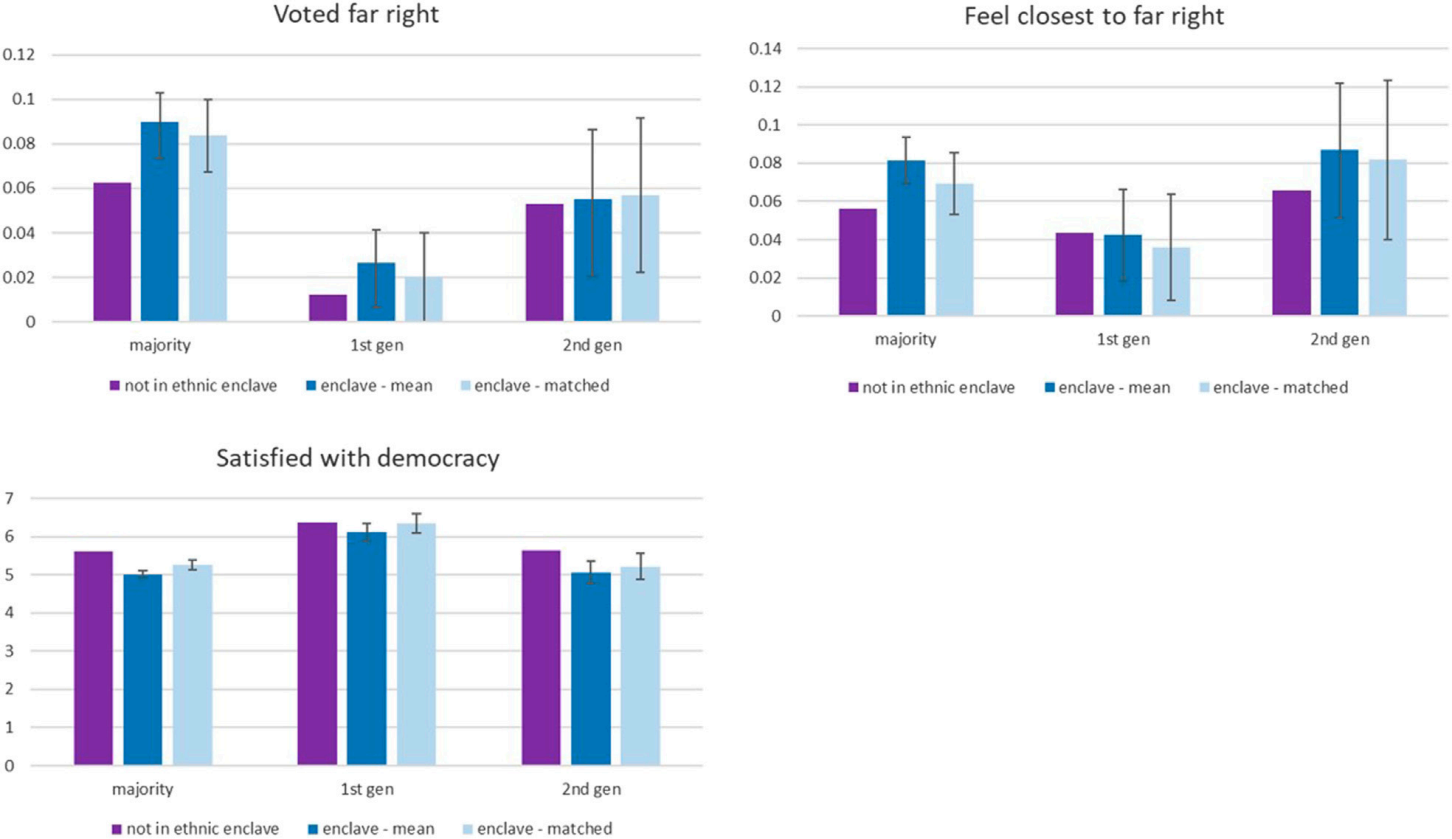
Voting behavior and satisfaction with democracy for inhabitants of an ethnic enclave and those living elsewhere—by migrant status. The mean difference is estimated through an independent samples *t*-test (two-tailed), and the matched difference shows the estimate after propensity score matching with five nearest neighbors, taking individual sociodemographic factors into account. Outcome variables voting behavior and satisfaction with democracy are examined and the difference between enclave and non-enclave residents reported.

Table 4 shows that after accounting for selection on observables, living in an ethnic enclave is significantly associated with the probability of having voted for a right-wing nationalist party in the last national elections and feeling close to such a party, and it diminishes satisfaction with democracy. Importantly, members of the majority who live in an ethnic enclave are 2 percentage points more likely to have voted for far-right parties in the last elections than their counterparts who do not live in such an enclave (compared to the average propensity of 6% for majority members living outside of an ethnic enclave, this constitutes a 40% increase)—Figure 2 demonstrates just how substantial this effect is on the average probability of voting for the far right. There is no corresponding association observed for migrants or the second generation. The association for the majority is slightly weaker (and not statistically significantly different from 0 or from the association for the second generation) when looking at whether they feel closest to a far-right party rather than to another party. Both majority and second-generation minority members residing in an ethnic enclave tend to be less satisfied with the state of democracy in their country than those who live in mixed or predominantly majority settings. Thus, we find strong evidence that residence in an enclave is associated with increased salience of oppositional political narratives on the part of majority members and with dissatisfaction with democracy for both majority and minority members.

**TABLE 4:** Difference in voting and political attitudes for inhabitants of ethnic enclaves and those in majority areas.

		Majority	First generation	Second generation
Vote far right (%)	Mean difference	2.7*** (0.66)	1.43* (0.76)	0.24 (1.58)
	Matched difference	2.09** (0.83)	0.79 (1.02)	0.4 (1.78)
	N treated | control	1559 | 14,494	378 | 990	253 | 1002
Feel close to far right (%)	Mean difference	2.52*** (0.63)	−0.11 (1.23)	2.11 (1.8)
	Matched difference	1.31 (0.82)	−0.74 (1.42)	1.59 (2.14)
	N treated | control	1559 | 14,494	378 | 990	253 | 1002
Left–right (0–10)	Mean difference	−0.59*** (0.05)	−0.25** (0.12)	−0.59*** (0.15)
	Matched difference	−0.35*** (0.06)	−0.02 (0.13)	−0.43** (0.17)
	N treated | control	2,409 | 21,348	562 | 1732	339 | 1462

*: *p* < 0.1; **: *p* < 0.05; ***: *p* < 0.01; The mean difference is estimated through an independent samples *t*-test (two-tailed), and the matched difference shows the estimate after propensity score matching with five nearest neighbors, taking individual sociodemographic factors into account.

#### Mediation: Conflict and Contact in Relation to Political Power

Table 5 shows the mediation effect of conflict, contact with minorities, and personal economic situation on political outcomes.^13^ Feelings of threat and conflict in the local area do give rise to some of these associations, but even when comparing inhabitants of an ethnic enclave to non-enclave residents with similar feelings of threat, many of the associations remain. Notably, accounting for employment differences does not dent the pattern for majority members—that is to say, it is not just those who are unemployed or in a low occupational position that are likely to vote for the far right. Accounting for conflict—economic insecurity and crime—reduces the gap in satisfaction with democracy substantially for the majority and for the second generation. For the latter, the difference is no longer statistically significant. Conflict seems then to be the major driver of the political dissatisfaction of the second generation in ethnic enclaves but not for the majority. Conflict and accounting of personal economic insecurity reduce the size of the estimate for the second generation, but it does not disappear.

**TABLE 5:** Conflict, contact, and employment status mediating the relationship between ethnic enclaves and voting and political behavior.

		Majority	First generation	Second generation
Vote far right (%)	Effect of ethnic enclave	2.09** (0.83)	0.79 (1.02)	0.4 (1.78)
	When accounting for conflict	2.21*** (0.83)	0.37 (1.33)	−0.95 (1.86)
	When accounting for contact	2.99*** (0.8)	1.96** (0.91)	0.87 (1.66)
	When accounting for employment	2.02** (0.83)	1.71* (0.88)	0 (1.9)
Feel close to far right (%)	Effect of ethnic enclave	1.31 (0.82)	−0.74 (1.42)	1.59 (2.14)
	When accounting for conflict	1.61** (0.81)	0.48 (1.21)	0.24 (2.05)
	When accounting for contact	1.61** (0.8)	1.22 (1.2)	1.98 (2.05)
	When accounting for employment	1.7** (0.8)	−0.21 (1.36)	2.09 (2.47)
Satisfaction with democracy (0–10)	Effect of ethnic enclave	−0.35*** (0.06)	−0.02 (0.13)	−0.43** (0.17)
	When accounting for conflict	−0.21*** (0.06)	−0.06 (0.13)	−0.26 (0.16)
	When accounting for contact	−0.34*** (0.06)	−0.14 (0.13)	−0.35** (0.16)
	When accounting for employment	−0.34*** (0.06)	−0.03 (0.13)	−0.33** (0.16)

Note: This shows the estimated difference between those living in an ethnic enclave and their counterparts who live in a more homogeneously majority area. People are matched on country and sociodemographic background. To account for conflict, we include feelings of unsafety, being a victim of crime, and regional unemployment rate. To account for contact, we include friendshipties with minority members and language skills. Employment includes a combined variable on being inactive, employed in low-quality jobs, employed in middle-status jobs, or employed in a high-status job. *: *p* < 0.1, **: *p* < 0.05, ***: *p* < 0.01.

## Conclusion

Using the 2002 and 2014 waves of the ESS, we find that, before we account for compositional differences, living in a perceived ethnic enclave is on average positively associated with the threat to economic power—activity, employment, and occupational status for all groups we examine. This is the case for majority members as well as for migrants and the second generation. Importantly, however, after matching on a rich set of individual variables to account for social selection, the employment enclave penalty disappears for majority members. However, living in the ethnic enclave is associated with poorer labor market outcomes for migrants and their descendants, and these findings are robust to many different specifications, including to unobserved characteristics. Lack of opportunity for contact seems the prime driver behind the enclave economic penalty of minorities (in terms of job quality) and migrants (in terms of activity and employment).

On the contrary, we have provided evidence that residing in an enclave appears to solidify the political concerns of majority members, and they are more likely (by 2 percentage points) to vote for far-right parties in such settings and to be dissatisfied with democracy. Such pattern of dissatisfaction with democracy is also notable among minority members.

This research has several limitations. First of all, we study the effect of the perceived composition of the ethnic enclave—unfortunately, we do not have information about the actual ethnic diversity of the local area. Additional tests we have performed and which are available in the Supplementary information show that there is an alignment between the ethnic composition of the region and the perceptions of ethnic heterogeneity captured in the ESS, which gives us some degree of confidence in the measure we have used. Second, it is possible that conflict arises between particular ethnic groups. Our approach does not allow for subtle differentiation between ethnic minority groups or indeed to reflect on increasing levels of xenophobia that some groups, Muslim groups in particular, may be exposed to (Allen, 2010; Marfouk, 2019). Further work should aim to explore these important aspects of enclave residence to better examine the postulates of the threat framework. Finally, we do not know the ethnic composition of the respondents’ workplaces or whether they reside and work in the same location. We nevertheless can comment on whether on average the diversity of the local area, in fact the substantial (perceived) presence of minorities, undermines the employment prospects of majority individuals—it does not.

Our study shows that in the European context, increased migrant and minority presence in the local area does not seem to be a viable economic threat for majority members, but ethnic enclaves and the isolation in them are associated with poorer employment prospects for both migrants and minorities. The interpretation of our results however aligns with observations of political commentators that majority members may experience their political power threatened and act to redress the balance by voting for a far-right party in such local areas. Exposure to the ethnic enclave may not only isolate the second generation from the occupational hierarchies of the mainstream labor market but also serve to consolidate their dissatisfaction with democracy. Notably, one's personal economic position does not seem to mediate our results for threat to political power, suggesting that the actual economic situation of the respondent is not the mechanism underlying this phenomenon.

## Data Availability

Information about the data and the archives through which it can be accessed can be find here: European Social Survey Round 1 Data (2002). Data file edition 6.5.NSD–Norwegian Centre for Research Data, Norway–Data Archive and distributor of ESS data for ESS ERIC; European Social Survey Round 7 Data (2014). Data file edition 2.1. NSD–Norwegian Centre for Research Data, Norway–Data Archive and distributor of ESS data for ESS ERIC. The original contributions presented in the study are included in the article/Supplementary Material, and further inquiries can be directed to the corresponding author.

## Footnotes

1 The parties are the SPO in Austria, Vlaams Blok/Vlaams Belang, and FN in Belgium. The ODS in Czechia, the Danish People’s Party in Denmark, True Finns in Finland, FN in France, Lega Nord in Italy, Progress Party in Norway, law and justice in Poland, National Renovator Party in Portugal, the List Pim Fortuyn and PVV in the Netherlands, the AfD and NPD in Germany, UKIP in the United Kingdom, and Sverigedemokrate in Sweden.

2 By partitioning the sample into three separate groups based on rough categories of country of birth of self and parents, we aim to compare like with like as much as the sample size allows. These groups are of course still very diverse and differ in composition across countries, which we address through the matching approach.

3 European Social Survey Round 1 Data (2002). Data file edition 6.5. NSD–Norwegian Centre for Research Data, Norway–Data Archive and distributor of ESS data for ESS ERIC.

4 European Social Survey Round 7 Data (2014). Data file edition 2.1. NSD–Norwegian Centre for Research Data, Norway–Data Archive and distributor of ESS data for ESS ERIC.

5 Greece, Italy, and Luxembourg were not involved in the 2014 round.

6 Respondents are asked: “How would you describe the area where you currently live?” While referring to people’s own perception of the neighborhood, the scale of this local area is unknown and may differ across respondents.

7 This still cannot account for selection on unobservable characteristics however, which is addressed more in the sensitivity tests.

8 Within the last year; 1–5 years ago; 6–10 years ago; 11–20 years ago; more than 20 years ago.

9 Unfortunately, the survey contains no local labor market identifiers at a lower level than the region. We therefore use regional employment rate as a proxy, following standard practice in the literature (Manacorda et al., 2012).

10 In 2002, respondents were asked if they have any close friends who are immigrants, and in 2014, they were asked whether they have any close friends who are of a different race or ethnic group from most people in the country. While these questions are different, they are included jointly to measure strong ties with minorities and are dichotomized to contrast having many minority/immigrant friends with having few or none.

11 The percentage bias for all covariates is substantially reduced and at acceptable thresholds, less than 5%. There is no longer any statistically significant (*p* < 0.1) relation between the matched covariates and the probability of living in an ethnic enclave after matching.

12 In results not shown here but available upon request, we also look at the association between living in an ethnic niche and our indicators of conflict or contact. Majority members who live in an ethnic enclave are also substantially more likely to report feeling unsafe when walking around at night and to have been a victim of burglary or assault in the last 5 years, consistent with previous studies (Glikman and Semyonov, 2012; Laméris et al., 2018). Residing in an ethnic enclave for migrants is associated with higher probability of feeling unsafe and with living in a region with a higher unemployment rate. Migrants residing in ethnic enclaves are also substantially more likely to have many minority friends. Second-generation individuals living in an ethnic enclave are more likely to be victims of crime and to feel unsafe similarly to majority members, and are much more likely to have many minority friends in enclave areas.

13 Personal economic situation is a combined variable with five categories: inactive, employed, working on a job in the lowest 25% of occupational status, working on a job in the middle 50% of occupational status, and working on a job in the top 25% of occupational status.
