# Supplementary material for: Ethnic Enclaves, Economic and Political Threat: An Investigation With the European Social Survey
Source: Front Sociol. 2021 Jul 6;6:660378. doi: 10.3389/fsoc.2021.660378 (PMC8290944; doi:10.3389/fsoc.2021.660378)
Supplement: Supplementary file 1 [file Table1.DOCX]

Supplementary Tables

# Supplementary Tables: Description of sample

Table A 1: Description of sample: mean and standard deviation

|  | Majority | Migrants | 2nd generation |
| --- | --- | --- | --- |
| Active | 0.9 (0.31) | 0.87 (0.34) | 0.9 (0.3) |
| Employed | 0.92 (0.27) | 0.87 (0.34) | 0.9 (0.3) |
| Occupational Status | 46.76 (18.9) | 42.5 (20.5) | 47.65 (18.94) |
| Vote far-right | 0.07 (0.25) | 0.02 (0.13) | 0.05 (0.23) |
| Feel close to far-right | 0.06 (0.23) | 0.04 (0.2) | 0.07 (0.25) |
| Satisfied with democracy | 5.55 (2.36) | 6.3 (2.39) | 5.53 (2.43) |
| Feel unsafe when walking in local area after dark | 0.16 (0.37) | 0.19 (0.39) | 0.2 (0.4) |
| Victim of burglary/assault last 5 years | 0.23 (0.42) | 0.23 (0.42) | 0.26 (0.44) |
| employment rate in regional area (centred) | -0.04 (5.25) | 0.16 (4.77) | 0.7 (3.97) |
| Several immigrant/minority friends | 0.13 (0.34) | 0.51 (0.5) | 0.3 (0.46) |
| A few immigrant/minority friends | 0.39 (0.49) | 0.34 (0.47) | 0.42 (0.49) |
| No immigrant/minority friends | 0.48 (0.5) | 0.15 (0.36) | 0.29 (0.45) |
| Speak country language at home | 0.98 (0.15) | 0.54 (0.5) | 0.93 (0.25) |
| year: 2014 | 0.42 (0.49) | 0.49 (0.5) | 0.48 (0.5) |
| Male | 0.49 (0.5) | 0.48 (0.5) | 0.49 (0.5) |
| Age | 41.3 (11.2) | 40.17 (10.26) | 39.25 (11.5) |
| Education: None/primary | 0.08 (0.27) | 0.11 (0.32) | 0.06 (0.23) |
| Education: Lower secondary | 0.16 (0.37) | 0.15 (0.35) | 0.14 (0.35) |
| Education: Upper secondary | 0.38 (0.49) | 0.34 (0.47) | 0.41 (0.49) |
| Education: Post-secondary non-tertiary | 0.05 (0.21) | 0.04 (0.2) | 0.05 (0.23) |
| Education: Tertiary | 0.33 (0.47) | 0.36 (0.48) | 0.34 (0.47) |
| Married | 0.56 (0.5) | 0.63 (0.48) | 0.49 (0.5) |
| Dependent child | 0.51 (0.5) | 0.58 (0.49) | 0.49 (0.5) |
| (very) poor health | 0.04 (0.19) | 0.05 (0.22) | 0.05 (0.21) |
| Domicile: Big city | 0.15 (0.36) | 0.32 (0.47) | 0.21 (0.41) |
| Domicile: suburbs | 0.15 (0.36) | 0.19 (0.39) | 0.18 (0.39) |
| Domicile: town/small city | 0.3 (0.46) | 0.28 (0.45) | 0.33 (0.47) |
| Domicile: village | 0.39 (0.49) | 0.22 (0.41) | 0.27 (0.45) |
| Parental education: Other | 0.04 (0.2) | 0.06 (0.23) | 0.06 (0.24) |
| Parental education: none/primary | 0.29 (0.46) | 0.31 (0.46) | 0.24 (0.43) |
| Parental education: lower secondary | 0.16 (0.37) | 0.13 (0.33) | 0.14 (0.35) |
| Parental education: upper secondary | 0.28 (0.45) | 0.22 (0.41) | 0.28 (0.45) |
| Parental education: Post-secondary non-tertiary | 0.03 (0.16) | 0.04 (0.19) | 0.03 (0.18) |
| Parental education: Tertiary | 0.2 (0.4) | 0.25 (0.43) | 0.24 (0.42) |
| Parental employment status: professional/managerial | 0.21 (0.41) | 0.24 (0.43) | 0.24 (0.43) |
| Parental employment status: intermediate | 0.45 (0.5) | 0.38 (0.49) | 0.4 (0.49) |
| Parental employment status: Semi-skilled/semi-routine | 0.15 (0.36) | 0.12 (0.33) | 0.12 (0.33) |
| Parental employment status: unskilled and routine | 0.14 (0.35) | 0.17 (0.38) | 0.14 (0.35) |
| Parental employment status: did not work | 0.05 (0.22) | 0.09 (0.28) | 0.1 (0.3) |
| Did not leave with both parents at age 14 | 0.04 (0.2) | 0.06 (0.24) | 0.05 (0.23) |
| At least one parent born in country |  | 0.09 (0.28) | 0.69 (0.46) |
| Years of residence: within last year |  | 0.02 (0.13) |  |
| Years of residence: 1-5 years |  | 0.17 (0.37) |  |
| Years of residence: 6-10 years |  | 0.2 (0.4) |  |
| Years of residence: 11-20 years |  | 0.26 (0.44) |  |
| Years of residence: More than 20 years |  | 0.36 (0.48) |  |

Table A2 shows the selection equation on living in an ethnic enclave. The probability reduces with age, is highest for the unmarried, those with lower qualifications, and in poor health (with the exception of the 2^nd^ generation). Inhabitants of a bit city or suburbs are more likely to live in an ethnic enclave. It is less likely for minority members who have at least one parent born in the country of residence.

Table A 2: Selection equation (odds ratios) for computation of propensity scores

|  | Majority | 1st generation | 2nd generation |
| --- | --- | --- | --- |
|  |  |  |  |
| Dummy: 2014 | 1.564*** | 1.951*** | 1.581*** |
|  | (0.0910) | (0.239) | (0.227) |
| Country (reference = AT) |  |  |  |
| BE | 0.816 | 1.129 | 0.914 |
|  | (0.209) | (0.485) | (0.148) |
| DE | 0.726 | 1.286 | 0.928 |
|  | (0.149) | (0.271) | (0.174) |
| DK | 0.588*** | 0.499*** | 0.412*** |
|  | (0.0886) | (0.111) | (0.111) |
| EL | 1.374 | 0.341*** | 1.410 |
|  | (0.288) | (0.0830) | (0.449) |
| ES | 1.305 | 0.990 | 2.171** |
|  | (0.240) | (0.402) | (0.672) |
| FI | 0.287*** | 0.368*** | 0.130*** |
|  | (0.0397) | (0.0952) | (0.0243) |
| FR | 1.820*** | 1.348 | 1.251 |
|  | (0.309) | (0.341) | (0.396) |
| IE | 0.697* | 1.118 | 0.732 |
|  | (0.142) | (0.322) | (0.360) |
| IT | 1.210 | 0.376 | 1.297 |
|  | (0.266) | (0.402) | (1.123) |
| LU | 0.502*** | 0.442*** | 0.735** |
|  | (0.0673) | (0.0733) | (0.0922) |
| NL | 0.709** | 1.303* | 1.139 |
|  | (0.0975) | (0.188) | (0.130) |
| NO | 0.640* | 0.585** | 1.038 |
|  | (0.147) | (0.126) | (0.560) |
| PT | 0.401** | 1.066 | 0.613*** |
|  | (0.184) | (0.390) | (0.111) |
| SE | 0.750* | 0.557** | 0.347*** |
|  | (0.127) | (0.151) | (0.132) |
| UK | 0.802 | 1.372 | 1.122 |
|  | (0.228) | (0.467) | (0.404) |
| Dummy: male | 0.796*** | 0.938 | 0.928 |
|  | (0.0330) | (0.0934) | (0.119) |
| Age | 0.988*** | 0.988** | 0.979*** |
|  | (0.00236) | (0.00551) | (0.00652) |
| Highest qualification (ref = upper secondary) |  |  |  |
| None/primary | 1.289** | 1.229 | 1.393 |
|  | (0.135) | (0.207) | (0.432) |
| Lower secondary | 1.170* | 1.143 | 1.109 |
|  | (0.100) | (0.138) | (0.200) |
| Post-secondary non-tertiary | 0.874 | 0.674 | 0.841 |
|  | (0.0933) | (0.179) | (0.255) |
| Tertiary | 0.732*** | 0.579*** | 0.853 |
|  | (0.0410) | (0.0789) | (0.153) |
| Dummy: married | 0.696*** | 1.031 | 0.654** |
|  | (0.0399) | (0.118) | (0.118) |
| Dummy: dependent child | 1.033 | 1.171 | 1.127 |
|  | (0.0529) | (0.127) | (0.170) |
| Dummy: poor health | 1.625*** | 1.729*** | 0.896 |
|  | (0.194) | (0.313) | (0.296) |
| Domicile (reference = town/small city) |  |  |  |
| Big city | 2.308*** | 1.778*** | 3.133*** |
|  | (0.229) | (0.237) | (0.432) |
| Suburbs of big city | 1.484*** | 1.568*** | 2.240*** |
|  | (0.163) | (0.184) | (0.409) |
| Rural | 0.337*** | 0.346*** | 0.439*** |
|  | (0.0346) | (0.0619) | (0.104) |
| Highest qualification parents (reference = upper secondary) | |  |  |
| Other | 0.326*** | 1.587** | 1.031 |
|  | (0.0769) | (0.349) | (0.449) |
| None/primary | 1.040 | 1.085 | 1.001 |
|  | (0.0950) | (0.184) | (0.201) |
| Lower secondary | 0.964 | 0.810 | 1.017 |
|  | (0.0817) | (0.159) | (0.176) |
| Post-secondary non-tertiary | 1.114 | 0.667 | 0.600 |
|  | (0.139) | (0.201) | (0.208) |
| Tertiary | 1.012 | 0.848 | 0.795 |
|  | (0.0964) | (0.142) | (0.198) |
| Employment status parent when aged 14 (reference = intermediate) |  |  |  |
| Professional/managerial | 0.966 | 1.125 | 1.013 |
|  | (0.0767) | (0.127) | (0.276) |
| Semi-skilled/semi-routine | 1.029 | 1.062 | 1.432 |
|  | (0.0925) | (0.196) | (0.353) |
| Unskilled and routine | 1.150* | 1.092 | 1.477** |
|  | (0.0850) | (0.134) | (0.290) |
| Did not work | 1.161 | 1.244 | 1.363 |
|  | (0.124) | (0.290) | (0.274) |
| Dummy: did not have both parents aged 14 | 1.174 | 1.085 | 1.604* |
|  | (0.116) | (0.183) | (0.428) |
| dummy: one parent born in country |  | 0.601*** | 0.734 |
|  |  | (0.113) | (0.150) |
| Years of residence (reference = 11-20 years) |  |  |  |
| Less than 1 year |  | 0.907 |  |
|  |  | (0.388) |  |
| 1-5 years |  | 0.968 |  |
|  |  | (0.155) |  |
| 6-10 years |  | 1.045 |  |
|  |  | (0.185) |  |
| More than 20 years |  | 0.998 |  |
|  |  | (0.127) |  |
| Constant | 0.258*** | 0.396*** | 0.430*** |
|  | (0.0414) | (0.136) | (0.125) |
|  |  |  |  |
| Observations | 24,159 | 2,579 | 1,831 |
| pseudo-r2 | 0.112 | 0.109 | 0.142 |

**** p<0.01, ** p<0.05, * p<0.1, Calculation of propensity score with data from ESS 2002 and 2014, showing odds ratio with standard errors clustered by regions*

Table A 3: Balance on covariates after matching

|  | **Majority** |  |  | **1st generation** | |  | **2nd generation** | |  |
| --- | --- | --- | --- | --- | --- | --- | --- | --- | --- |
| **Variable** | **Treated** | **Control** | **%bias** | **Treated** | **Control** | **%bias** | **Treated** | **Control** | **%bias** |
|  |  |  |  |  |  |  |  |  |  |
| Dummy: 2014 | 0.50 | 0.50 | 0.7 | 0.62 | 0.63 | -1.3 | 0.57 | 0.59 | -3.1 |
| BE | 0.05 | 0.05 | -1 | 0.09 | 0.09 | 0 | 0.10 | 0.09 | 3.4 |
| DE | 0.10 | 0.10 | 0.6 | 0.13 | 0.13 | 0.7 | 0.14 | 0.14 | 2 |
| DK | 0.05 | 0.05 | 0.5 | 0.03 | 0.03 | -0.8 | 0.02 | 0.02 | 0.3 |
| EL | 0.08 | 0.08 | -1 | 0.03 | 0.03 | -1.2 | 0.04 | 0.04 | -2.3 |
| ES | 0.12 | 0.12 | -0.5 | 0.06 | 0.07 | -4.7 | 0.03 | 0.03 | -2.8 |
| FI | 0.03 | 0.03 | 1.6 | 0.01 | 0.02 | -1.1 | 0.00 | 0.01 | -4.6 |
| FR | 0.09 | 0.08 | 4 | 0.09 | 0.09 | 0.2 | 0.14 | 0.14 | 2.2 |
| IE | 0.07 | 0.07 | -0.3 | 0.08 | 0.07 | 2 | 0.03 | 0.03 | 1.7 |
| IT | 0.03 | 0.04 | -1.8 | 0.00 | 0.00 | -0.6 | 0.01 | 0.01 | 0 |
| LU | 0.00 | 0.01 | -0.6 | 0.03 | 0.04 | -0.2 | 0.03 | 0.04 | -3.6 |
| NL | 0.07 | 0.07 | -0.5 | 0.10 | 0.08 | 4.3 | 0.09 | 0.09 | 1.5 |
| NO | 0.06 | 0.07 | -2.6 | 0.03 | 0.03 | 0.5 | 0.03 | 0.04 | -2.5 |
| PT | 0.04 | 0.03 | 0.4 | 0.03 | 0.03 | -2.7 | 0.01 | 0.01 | 1.9 |
| SE | 0.05 | 0.05 | -1.7 | 0.09 | 0.08 | 1.6 | 0.05 | 0.05 | -0.2 |
| UK | 0.06 | 0.06 | 2.1 | 0.09 | 0.09 | 0.4 | 0.13 | 0.13 | -0.7 |
| Dummy: male | 0.44 | 0.44 | 0.2 | 0.48 | 0.48 | -0.2 | 0.48 | 0.48 | -0.1 |
| Age | 39.54 | 39.63 | -0.7 | 39.66 | 39.80 | -1.4 | 36.29 | 36.94 | -5.6 |
| None/primary | 0.09 | 0.09 | 1.6 | 0.14 | 0.14 | -1 | 0.07 | 0.07 | 2.4 |
| Lower secondary | 0.18 | 0.17 | 0.7 | 0.17 | 0.17 | 0.3 | 0.17 | 0.18 | -3.2 |
| Post-secondary non-tertiary | 0.05 | 0.05 | -0.5 | 0.04 | 0.03 | 1.7 | 0.05 | 0.04 | 1.6 |
| Tertiary | 0.30 | 0.32 | -3.1 | 0.29 | 0.29 | 0.1 | 0.31 | 0.32 | -1.2 |
| Dummy: married | 0.44 | 0.44 | 0.4 | 0.62 | 0.63 | -2.1 | 0.37 | 0.37 | -0.1 |
| Dummy: dependent child | 0.46 | 0.46 | 0.3 | 0.61 | 0.62 | -2.8 | 0.43 | 0.42 | 2.4 |
| Dummy: poor health | 0.06 | 0.05 | 2.1 | 0.07 | 0.07 | 0.6 | 0.05 | 0.05 | 0.5 |
| Big city | 0.33 | 0.33 | 0.3 | 0.43 | 0.44 | -1.1 | 0.40 | 0.42 | -3 |
| Suburbs of big city | 0.20 | 0.21 | -2.7 | 0.21 | 0.21 | 0.6 | 0.23 | 0.23 | 0 |
| Rural | 0.15 | 0.15 | 2 | 0.09 | 0.09 | -1.2 | 0.10 | 0.11 | -2.7 |
| Other | 0.01 | 0.01 | -0.2 | 0.05 | 0.05 | 0.8 | 0.02 | 0.02 | 1.4 |
| None/primary | 0.31 | 0.31 | 0.8 | 0.36 | 0.39 | -4.6 | 0.31 | 0.31 | -1.6 |
| Lower secondary | 0.15 | 0.16 | -0.4 | 0.12 | 0.12 | -0.1 | 0.16 | 0.16 | -0.5 |
| Post-secondary non-tertiary | 0.03 | 0.03 | -1.9 | 0.03 | 0.02 | 2 | 0.03 | 0.02 | 2.3 |
| Tertiary | 0.21 | 0.21 | 0 | 0.22 | 0.22 | 0.7 | 0.21 | 0.23 | -4.1 |
| Professional/managerial | 0.21 | 0.20 | 0.8 | 0.21 | 0.21 | 1.9 | 0.20 | 0.21 | -1.8 |
| Semi-skilled/semi-routine | 0.14 | 0.15 | -2.5 | 0.12 | 0.12 | -0.1 | 0.14 | 0.14 | 0.7 |
| Unskilled and routine | 0.14 | 0.14 | 1.3 | 0.19 | 0.20 | -1.9 | 0.17 | 0.18 | -2.4 |
| Did not work | 0.07 | 0.07 | -1 | 0.11 | 0.12 | -4.1 | 0.14 | 0.14 | 2.2 |
| Dummy: did not have both parents aged 14 | 0.05 | 0.05 | 1.6 | 0.07 | 0.07 | 2.4 | 0.08 | 0.09 | -5.7 |
| At least one parent born in country |  |  |  | 0.05 | 0.06 | -0.7 | 0.57 | 0.58 | -2.2 |
| Less than 1 year |  |  |  | 0.02 | 0.02 | 0 |  |  |  |
| 1-5 years |  |  |  | 0.15 | 0.15 | 0.7 |  |  |  |
| 6-10 years |  |  |  | 0.19 | 0.18 | 3.4 |  |  |  |
| More than 20 years |  |  |  | 0.36 | 0.35 | 2.9 |  |  |  |

*Description of covariate balancing before and after matching on 5 nearest neighbours, showing the average before and after matching, and the % bias. None of the differences are statistically significant after matching.*

### Sensitivity to unobserved confounder

While the propensity score matching accounts for selection on observed characteristics, there may be selection on unobserved characteristics such as motivation or preferences which can affect both the probability of living in an ethnic enclave and outcomes; and thereby bias the estimated effect. We test the robustness of these results to three simulated unobserved binary confounders mimicking the relations of three strong confounders: having tertiary qualifications; feeling unsafe when walking in the local area at night; and a self-reported measure on whether the household struggles financially. Tables A1 and A2 show the difference in employment (A1) and voting far-right (A2) between inhabitants of an ethnic enclave and others before (prior) and after (post) including the simulated unobserved confounder. It also shows the association, in odds ratio, between the confounder and living in an enclave (selection effect) and employment (outcome effect). This test indicates that the lower employment rate of majority inhabitants of ethnic enclaves is not very robust, while the association between labour market outcomes and living in an ethnic enclave for migrants and the 2^nd^ generation remains quite similar. Including an indicator as strongly related to both living in an ethnic enclave and employment as struggling financially reduces the association most, but it remains well within the bounds of error for the reported effect for migrants and the 2^nd^ generation. Similarly, the association of voting for far-right wing parties when living in an ethnic enclave for the majority remains quite similar even when including strong unobserved confounders.

Table A 4: Sensitivity of including unobserved confounder on the difference in employment by ethnic enclave

| **Employed** |  | Majority | Migrant | 2^nd^ generation |
| --- | --- | --- | --- | --- |
| **Prior effect (ATT)** | | -1.9* (1.0) | -6.2** (2.5) | -3.4 (3.5) |
| **Feeling unsafe** | Selection effect | 3.072 | 0.528 | 0.667 |
|  | Outcome effect | 0.672 | 2.642 | 2.409 |
|  | ATT post | -0.8 (1.4) | -6.2* (3.2) | -3.2 (4.4) |
| **Struggle financially** | Selection effect | 0.151 | 0.16 | 0.189 |
|  | Outcome effect | 1.771 | 1.671 | 2.208 |
|  | ATT post | 0.00 (1.3) | -5 (3.3) | -1.7 (4.4) |
| **Degree** | Selection effect | 2.377 | 1.542 | 2.913 |
|  | Outcome effect | 0.909 | 0.686 | 0.907 |
|  | ATT post | -1.4 (1.2) | -6.9** (3.1) | -4.4 (4) |

**:p<0.1; **:p<0.05; ***:p<0.01, estimated ATT after nearest neighbour matching and including an unobserved confounder, mimicking the association of three existing variables (having a degree, feeling unsafe, or struggling financially) with living in an ethnic enclave: (selection) and employment (outcome),*

Table A 5: Sensitivity of including unobserved confounder on the difference in voting far-right by ethnic enclave

| **Vote right** |  | Native | Migrant | 2^nd^ gen |
| --- | --- | --- | --- | --- |
| **Prior effect (ATT)** | | 2.8*** (0.9) | 1.96** (0.9) | 1.2 (2.4) |
| **Feeling unsafe** | Selection effect | 1.291 | 3.278 | 2.738 |
|  | Outcome effect | 3.313 | 2.974 | 2.918 |
|  | ATT post | 2.1 (1.3) | 1 (1.4) | -0.7 (3.1) |
| **Struggle financially** | Selection effect | 0.991 | 5.701 | 1.593 |
|  | Outcome effect | 1.857 | 2.678 | 2.137 |
|  | ATT post | 2.4* (1.3) | 0.9 (1.5) | 0 (3.2) |
| **Degree** | Selection effect | 0.376 | 0.314 | 0.452 |
|  | Outcome effect | 0.926 | 0.504 | 0.888 |
|  | ATT post | 2.3* (1.2) | 1 (1.4) | 0.1 (3) |

**:p<0.1; **:p<0.05; ***:p<0.01, estimated ATT after nearest neighbour matching and including an unobserved confounder, mimicking the association of three existing variables (having a degree, feeling unsafe, or struggling financially) with living in an ethnic enclave: (selection) and employment (outcome).*

### Sensitivity to different specifications

We vary the specifications through which the treatment effect is estimated, shown in table A6. First, we match on contextual factors by including the average employment rate, share of workers in low-skilled occupations; share of the primary and secondary industrial sector; and the share of migrants and minorities in the region, in the selection equation to account for regional differences. This means we then estimate the effect of living in an ethnic enclave not only for otherwise similar residents, but also living within comparable regions. Second, we account for pan-ethnic characteristics by including the share of minorities of the same continental origin group, and the share of graduates and recent migrants within this pan-ethnic community, at the regional level. To account for the economic and cultural distance for migrants we include GDP, female labour force participation, the share of tertiary enrolment in the country and the share of non-Christians^[[1]](#footnote-1)^ in the sending country. This means we estimate the effect among comparable migrants and 2^nd^ generation individuals regarding their community support and origin characteristics. Third, we change the definition of an ethnic enclave to an area with some or many minorities rather than (almost) none. Fourth, we use a more restrictive control group by comparing the inhabitants of an ethnic enclave with those who live in an area where they report almost no minorities living, rather than also including areas with some minorities. Fourth, in order to ensure the inhabitants of ethnic enclave are as close to those living elsewhere as possible we also match on two human values indicators from the Schwarz scales: stimulation and achievement. Fifth, we restrict the sample to the 2014 wave. Finally, the matching is done separately for respondents living in a big city in the capital region and the rest to test whether there is a difference in cosmopolitan cities.

While there is some variation the effects of the majority are relatively robust. There is a somewhat more negative effect on activity in capital cities than outside; a more negative employment effect when only looking at 2014; and there is some evidence of a lower occupational status when living in an ethnic enclave if we match on regional characteristics and on human values, or outside of the capital. For 1^st^ generation migrants we notice that negative effects of living in an ethnic niche on activity and employment seem to be worse in 2014 than in 2002. Effects are also worse when comparing an ethnic enclave to an area with few minorities, particularly for occupational status. The main findings remain quite constant though. For the 2^nd^ generation there is some variation between the capital city and outside of the capital city.

Table A 6: Sensitivity of treatment effect to different specifications

|  |  | **Majority** | **Migrant** | **2nd generation** |
| --- | --- | --- | --- | --- |
| Active (%) | Base match | -0.22 (0.72) | -3.12* (1.76) | -2.32 (1.65) |
|  | Regional | -0.6 (0.73) | -3.3* (1.84) | -2.72 (1.75) |
|  | Community/sending | -0.22 (0.72) | -2.85 (1.88) | -0.53 (1.7) |
|  | Restricted minority | -0.62 (0.92) | -5.3** (2.14) | -2.78 (2.17) |
|  | Human values | -0.45 (0.73) | -4.08** (1.77) | -2.84 (1.77) |
|  | 2014 | 1.41 (0.93) | -9*** (1.9) | -2.55 (1.8) |
|  | Capital city | -3.3*** (1.24) | -3.83 (3.74) | -6.88** (2.68) |
|  | Not in capital city | 0.52 (0.87) | -3.62* (2.14) | -2.06 (1.97) |
| Employed (%) | Base match | -1.68** (0.8) | -6.94*** (2.04) | -4.58 (2.86) |
|  | Regional | -0.9 (0.81) | -7.67*** (2.07) | -5.42* (2.81) |
|  | Community/sending | -1.68** (0.8) | -7.02*** (2.07) | -2.93 (3.11) |
|  | Restricted minority | -1.55 (0.94) | -7.78** (3.31) | -0.9 (3.29) |
|  | Human values | -1.92** (0.81) | -8.02*** (2.07) | -3.2 (2.73) |
|  | 2014 | -3*** (1.13) | -8.14*** (2.82) | -2.48 (3.11) |
|  | Capital city | -0.55 (1.62) | -8.63*** (2.84) | -0.23 (6.97) |
|  | Not in capital city | -1.87** (0.94) | -6.97*** (2.59) | -5.71* (3.25) |
| Occupational status | Base match | -0.63 (0.44) | -1.27 (1.04) | -2.56** (1.24) |
|  | Regional | -1.36*** (0.47) | -0.77 (1.07) | -5.38*** (1.25) |
|  | Community/sending | -0.63 (0.44) | -0.99 (1.07) | -2.86** (1.32) |
|  | Restricted minority | -0.33 (0.52) | -5.22*** (0.9) | -6.19*** (1.68) |
|  | Human values | -0.91** (0.45) | -0.66 (1.13) | -2.97** (1.45) |
|  | 2014 | -0.54 (0.71) | -0.45 (1.48) | -2.59* (1.51) |
|  | Capital city | -0.23 (0.84) | 0.1 (1.95) |  |
|  | Not in capital city | -0.94* (0.51) | -2.06 (1.32) | -3.5** (1.38) |

*Estimated treatment effect of living in an ethnic enclave on being active, employed and occupational status, using different specifications *:p<0.1; **:p<0.05; ***:p<0.01*

***Supplementary Figures***

Figure A1: Share of minorities and perception of living in area with many migrants and minorities

Figure A1 Comes about here

*Source: LFS 2008 and 2014 for share of migrants and the 2^nd^ generation; ESS 2002 and 2014 for average of respondents saying they live in a perceived ethnic enclave.*

1. Data on the GDP per capita, female labour force participation and tertiary enrolment rate in the sending country are measured as the average in the sending country between 1990 and 2014, obtained from the World bank on 10/02/2017: <http://data.worldbank.org/indicator/SL.TLF.CACT.FE.ZS>. The religious information is obtained from the ARDA world religion dataset (Maoz and Henderson, 2013). [↑](#footnote-ref-1)
